# A Milestone in Multiple Sclerosis Therapy: Monoclonal Antibodies Against CD20—Yet Progress Continues

**DOI:** 10.1007/s13311-021-01048-z

**Published:** 2021-04-20

**Authors:** Esther S. Frisch, Roxanne Pretzsch, Martin S. Weber

**Affiliations:** 1 Institute of Neuropathology, University Medical Center, Georg August University, 37099 Göttingen, Germany; 2 Department of Neurology, University Medical Center, Georg August University, 37099 Göttingen, Germany

**Keywords:** Anti-CD20 treatment, B cells in MS, Ocrelizumab, Rituximab, Ublituximab, Ofatumumab

## Abstract

Multiple sclerosis (MS), which is a chronic inflammatory disease of the central nervous system, still represents one of the most common causes of persisting disability with an early disease onset. Growing evidence suggests B cells to play a crucial role in its pathogenesis and progression. Over the last decades, monoclonal antibodies (mabs) against the surface protein CD20 have been intensively studied as a B cell targeting therapy in relapsing MS (RMS) as well as primary progressive MS (PPMS). Pivotal studies on anti-CD20 therapy in RMS showed remarkable clinical and radiological effects, especially on acute inflammation and relapse biology. These results paved the way for further research on the implication of B cells in the pathogenesis of MS. Besides controlling relapse development in RMS, ocrelizumab (OCR) also showed clinical benefits in patients with PPMS and became the first approved drug for this disease course. In this review, we provide an overview of the current anti-CD20 mabs used or tested for the treatment of MS—namely rituximab (RTX), OCR, ofatumumab (OFA), and ublituximab (UB). Besides their effectiveness, we also discuss possible limitations and safety concerns especially in regard to long-term treatment, both for this class of drugs overall as well as for each anti-CD20 mab individually. Additionally, we elucidate to what extent anti-CD20 therapy may alter the function of other immune cells, both directly or indirectly. Finally, we cover the current knowledge on repopulation of CD20^+^ cells after cessation of anti-CD20 treatment and discuss future aspirations towards alternative, further developed B cell silencing therapies.

**Supplementary Information:**

The online version contains supplementary material available at 10.1007/s13311-021-01048-z.

## Introduction

Multiple sclerosis (MS) is an inflammatory disease of the central nervous system (CNS). The most common early course is relapsing–remitting MS (RRMS), which is characterized by intermittent exacerbations that are followed by periods of complete or partial recovery. RRMS can transition into the secondary progressive form (SPMS). The term relapsing MS (RMS) unites RRMS and SPMS with superimposed relapses. The primary progressive course (PPMS) is defined by continuous deterioration of disability, independent of relapses. In order to categorize the respective disease course precisely, the new classification by Lublin et al. [1] should be applied. The term disability worsening is currently used to describe a stepwise clinical deterioration associated with RMS. In contrast, disability progression describes a continuous accumulation of disability linked to the progressive forms of MS. However, patients with RMS frequently accumulate disability progression even though relapse activity appears to be well controlled. Therefore, Kappos et al. [2] introduced the concept of confirmed disability accumulation (CDA) subdivided into relapse associated worsening (RAW) and progression independent from relapse activity (PIRA). RAW is classified as a deterioration of disability occurring within 90 days after relapse, while PIRA develops temporally independently of relapses [2]. This new concept stands independent from the clinical distinction of RMS and PPMS and provides the ability to describe and distinguish individual courses more precisely.

For a long time, T cells were seen as the key effector cells in MS. However, new evidence highlights the crucial role of B cells in the pathogenesis of MS. As B cells have three main immunological functions, they are assumed to be involved in the pathogenesis of MS in different ways.

First, they act as very potent antigen presenting cells (APC). B cells recognize antigens with their B cell receptor (BCR) and initiate T cell responses. In MS, encephalitogenic T cells are thought to be activated by B cells and their interaction is decisive when only small amounts of antigen are present [3]. For the activation of T cells, B cells express different co-stimulatory molecules such as CD40, CD80, and CD86 on their surface [4]. Interestingly, the co-stimulatory molecule CD40 as well as major histocompatibility complex class II (MHCII) was found to be more highly expressed in MS patients than in healthy controls [5].

Second, B cells produce cytokines and are therefore able to regulate immune responses in a pro- and anti-inflammatory way. Studies demonstrate that B cells of patients diagnosed with MS are chronically activated and show an alteration of their cytokine profile. They are matured in a pro-inflammatory manner resulting in a higher production of IL-6 compared to B cells of healthy individuals [6–8]. When stimulated in vitro via a two-signal model of activation through the BCR and CD40, B cells of MS patients produce more lymphotoxin (LT) and tumor necrosis factor α (TNFα) compared to healthy controls, too [8]. In experimental autoimmune encephalomyelitis (EAE)—an animal model of MS—it was demonstrated that IL-6 derived from B cells induces pathogenic Th17 differentiation of T cells and inhibits the development of regulatory T cells (T_reg_) [9, 10]. These findings suggest that chronically activated B cells in MS foster the maturation of encephalitogenic Th17 cells. Furthermore, a study by Fillatreau and colleagues [11] suggests that IL-10-producing B cells have a regulatory impact on autoimmunity. Studies indicate that the anti-inflammatory cytokine IL-10 is predominantly produced by naïve B cells, while TNFα and LT are largely produced by differentiated memory B cells [6, 12]. However, this observation is most likely context dependent [12]. Importantly, B cells from MS patients were found to express lower levels of IL-10, assuming a dysregulation of the immune system [12].

Third, B cells are precursors of antibody-secreting plasma cells and therefore also the source of potentially pathogenic antibodies. Although the occurrence of antibodies directed against myelin oligodendrocyte glycoprotein (MOG) and aquaporin-4 (AQP4) is now considered to be own entities (reviewed by Fujihara et al. [13]), the existence of dysregulated humoral immune activity in MS has been known ever since abnormal production of intrathecally synthesized immunoglobulin (Ig) G in forms of oligoclonal bands (OCBs) was described in the 1940s [14]. While it remains unclear whether OCBs contribute to the pathogenesis and progression of MS [15], their presence is considered evidence for dissemination in time according to the current version (2017 revisions) of the McDonald criteria for the diagnosis of MS [16]. The contribution of B cells to the pathogenesis of MS is further underlined by the presence of B cells in MS lesions. On the basis of brain biopsies and autopsies of MS patients, four different histopathological patterns of demyelinating lesions were described. The most common pattern II contains Igs, indicating antibody involvement in demyelinating lesions [17].

Within the CNS of MS patients, B cells are found not only in brain parenchyma but also in the meningeal compartment [17, 18]. Cerebral meninges of mainly SPMS patients contain lymphoid follicle-like structures, comparable to ectopic lymphoid tissue (eLT) found in autoimmune diseases like rheumatoid arthritis (RA), Sjörgren syndrome, or autoimmune thyroiditis [18, 19]. Magloizzi et al. [20] demonstrated the presence of germinal center formation within these lymphoid follicle-like structures, representing a site of T cell regulated differentiation of B cells into antibody-secreting cells or memory B cells. Meningeal eLFs are seen as a potential source of pathogenic B and T cell responses and are, therefore, discussed as presumptive drivers of progression of MS (reviewed by Negron et al. [21]). While the possible role of lymphoid follicle-like structures in the pathogenesis of SPMS remains unclear, it was found that their presence correlates with an early onset of disease, an early irreversible disability, and death at younger age. Moreover, studies indicate that SPMS with meningeal follicles is characterized by a more severe cortical pathology compared to SPMS without meningeal follicles [19, 20].

Due to the abovementioned, growing evidence of an implication of B cells to the pathogenesis of MS, B lymphocytes were identified as a possible target for MS therapy. In 2008, Bar-Or and colleagues [22] published first results of anti-CD20 depletion in MS patients. CD20 is expressed on the cell surface of a range of B lymphocytes including immature B cells—starting from the pre-B cell stage—and mature B cells. However, it is lost on terminal differentiated plasma cells. Interestingly, CD20 can also be found on a subset of T cells [23–25]. Hence, monoclonal antibodies (mab) against CD20 deplete immature B cells and mature B cells as well as CD20^+^ T cells, but spare CD20-negative plasma cells.

The relevant anti-CD20 antibodies regarding MS therapy are rituximab (RTX), ocrelizumab (OCR), ublituximab (UB), and ofatumumab (OFA). These four antibodies all belong to the group I CD20 antibodies. This means that the depletion is mediated through complement-dependent (CDC) and antibody-dependent cellular cytotoxicity (ADCC) [26]. RTX was originally developed for the treatment of non-Hodgkin’s B cell lymphoma and became the first anti-CD20 antibody to be approved for this indication by the FDA in 1997. In 2006, RTX gained approval for the treatment of RA refractory to TNFα-blocking agents. Shortly after, it was tested in an open-label phase I trial on a small cohort of 26 patients with RRMS [22]. In 2008, Hauser et al. [27] published the promising results of a phase II study on patients with RRMS (HERMES trial). This study was followed by a trial testing RTX in PPMS patients (OLYMPUS trial) but failed to meet its primary endpoint [28]. RTX never reached approval for MS as among other things, the attention was drawn to the further humanized antibody OCR. The results of two phase III trials on OCR (OPERA I and II) led to approval by the FDA for treatment of RRMS and PPMS in 2017 [29, 30]. Shortly thereafter, the fully humanized anti-CD20 antibody OFA, which can be administered subcutaneously (s.c.) or intravenously (i.v.), was tested in two phase III clinical trials (ASCLEPIOS I and II) [31]. It gained approval for the treatment of RRMS, secondary progressive multiple sclerosis (SPMS), and clinically isolated syndrome through the FDA in August 2020. OFA is the first and only self-administered B cell depleting therapy for the treatment of MS. The chimeric antibody UB is glycoengineered and differs slightly from the other abovementioned anti-CD20 antibodies regarding its mode of action. It is currently being tested for the treatment of MS in phase III (ULTIMATE I and II) studies.

In this article, we give an overview of the different anti-CD20 antibodies used in the treatment of MS and discuss the advantages and the unsolved problems regarding the approach of depleting CD20^+^ cells.

## Rituximab

The chimeric antibody (IgG1) RTX was firstly approved by the FDA for the treatment of non-Hodgkin’s B cell lymphoma in 1997 [32, 33]. It mediates the depletion of CD20^+^ cells predominantly via CDC and, to a smaller extent, through ADCC [34]. RTX is administered i.v. and is capable of crossing the blood–brain barrier (BBB). Although only a small proportion of 0.1% of plasma concentration reaches the CNS, it effectively reduces B cells in the cerebrospinal fluid (CSF) and hereby leads to a reduction of T cells in the CSF, too [35, 36].

### Phase II

In 2008, Hauser et al. [27] published the results of a phase II trial (HERMES) in which one course of RTX was administered i.v. to patients with relapsing–remitting multiple sclerosis. The double-blind 48-week trial included 104 patients. Thereof, 69 patients received 1000 mg of RTX on days 1 and 15, while 35 patients received placebo infusions. As the primary endpoint, the number of gadolinium-enhancing lesions (GELs) on T_1_-weighted MRI brain scans at weeks 12, 16, 20, and 24 was determined. Moreover, the proportion of patients with relapses, the annualized rate of relapse, the total number of new GELs observed in the MRI brain scans as well as change from the baseline lesion volume on T_2_-weigthed MRI scans was measured. The baseline lesion volume was determined 4 weeks before the first infusion. The amount of GELs and newly developed GELs was significantly lower in the RTX receiving group at all times of measurement. Thus, the study met its primary endpoint. The relative reduction of GELs in the RTX-treated group was 91%. Moreover, in comparison to the placebo group, the proportion of patients in the RTX group who experienced relapses was reduced in weeks 24 and 48. The treatment was associated with a decline in the volume of lesions on T_2_-weighted MRI scan from baseline to weeks 24 and 36.

### RTX in PPMS

While most MS patients experience clinical relapses, 10 to 15% of all MS patients have PPMS. It is considered to have a similar pathogenesis; however, there are some differences. In a different phase II/III trial (OLYMPUS), the effect of four courses of RTX on patients diagnosed with PPMS was examined. The results were published by Hawker et al. [28] in 2009. Four hundred thirty-nine patients diagnosed with PPMS were assigned to either the RTX or the placebo group in a 2:1 ratio. They received i.v. 1000 mg RTX or placebo infusions on study weeks 0, 2, 24, 26, 48, 50, 72, and 74. Confirmed disease/disability progression (CDP) was determined as the primary endpoint and defined as a rise in EDSS that maintained for at least 12 weeks. Changes in the total brain volume in the MRI scan in comparison to the baseline and the lesions’ volume in the T_2_-weighted MRI scan were examined as secondary endpoints. The primary endpoint was also analyzed in prespecified subgroups, for instance according to the patients’ age, gender, or the presence of GELs in the screening period before treatment. The study did not meet its primary endpoint, as the CPD was not delayed significantly (*p* = 0.1442) in the RTX group. Regarding the secondary endpoints, the increase of T_2_ lesion volume was significantly lower in the RTX group, while the decrease of brain volume was similar in both groups. Furthermore, analysis of the predefined subgroups showed that the CDP was delayed in RTX-treated patients who were younger than 51 years or had baseline GELs. The difference from placebo receiving patients was even higher for patients who were < 51 years old and showed active CNS inflammation in the screening period. Nevertheless, it should be noted that no benefit and even a debatable trend towards deterioration of the disease course was observed for patients who were ≥ 51 years old and had no GEL in the baseline measurement. These findings suggest that age and existence of GELs might be predictors for success of RTX treatment in patients with PPMS and paved the way for trials on other anti-C20 antibodies in PPMS.

### Retrospective Studies

In 2016, the results of a retrospective uncontrolled observational study that gathered data on off-label RTX-treated MS patients were published. The clinical data of 822 patients, either diagnosed with RRMS, PPMS, or SPMS, were analyzed. The patients received 500 or 1000 mg RTX i.v. every 6–12 months for a mean period of 22 months. The study primarily aimed to examine the safety of RTX in MS, but also investigated its efficacy on clinical and MRI measures. The annual relapse rate was 0.044 for patients with RRMS, 0.038 for SPMS, and 0.015 for PPMS. This study indicates with level IV evidence that RTX is safe and effective for the treatment of MS for up to 2 years [37].

## Ocrelizumab

After first striking results, a new partly humanized anti-CD20 antibody was developed by Roche [30]. OCR represents a second-generation anti-CD20 mab, possessing a partly human IgG1 tail and sharing some common epitope with its prototype, RTX [38]. Like RTX, it targets the large extracellular loop of CD20. However, it is expected to have a better benefit-to-risk profile due to its less immunogenic humanized IgG1 tail. OCR was first tested in patients with RRMS in a large phase II study that compared two different doses and offered promising results [39, 40]. Follow-up studies were performed (OPERA I and II) for RRMS as well as for PPMS (ORATORIO). Based on the convincing results of these ground-breaking studies, OCR was approved for adults with RMS and active disease by the FDA in March 2017 and shortly afterwards by the European Medicines Agency (EMA) in January 2018 [30]. Because of significant results in patients with PPMS, OCR became the first approved and long-awaited drug that was proven to diminish both clinical and MRI disease activity in patients with the primary progressive form of this disease.

### Phase II

OCR was tested in a multicenter, randomized, double-blinded, placebo-controlled phase II trial in order to determine an adequate dose. Two hundred twenty RMS patients, aged 18 to 55 years, were randomly allocated to four different groups: OCR 600 mg i.v., OCR 2000 mg i.v., interferon beta-1a (IFN-β1a) 30 µg subcutaneous, and placebo. OCR 600 mg i.v. showed the best benefit-to-risk profile with a relative reduction of 89% GELs in T_1_-weighted MRI at week 24 [39]. In an open-label extension phase (OLE) of the study, the positive results of this trial, namely minimal MRI activity and reduced annualized relapse rate, were reported for both OCR doses and also after 144 weeks of treatment [40].

### Phase III

After identifying the adequate dose in a phase II trial, OCR 600 mg i.v. was tested in two identical, double-blinded, placebo-controlled phase III studies (OPERA I and II) including a total number of 1656 RMS patients, aged between 18 and 55 years. All patients had experienced at least two clinical, documented relapses within 2 years or one within the prior year before recruiting. Their mean EDSS was 2.8. Patients were randomly allocated to either (a) 600 mg OCR i.v. every 24 weeks or (b) 44 µg IFN-β1a s.c. three times a week. The trials were conducted over 96 weeks, and the primary endpoint was the annualized relapse rate. Secondary endpoints were, among others, the mean number of GELs in MRI, the hazard of disability progression, and the percentage of patients reaching no evidence of disease activity (NEDA) criteria.

The efficacy of OCR in patients with RMS was confirmed in both studies: OCR proved to lower annualized relapse rates by 46% (OPERA I) and 47% (OPERA II) compared to IFN-β1a. The mean number of GELs was significantly lower with OCR (OPERA I 94%, OPERA II 95%). Furthermore, the proportion of patients with disability progression was significantly reduced under OCR treatment at weeks 12 and 24, while the percentage of patients meeting NEDA criteria increased from 29.2 to 47.9% in OPERA I and from 25.1 to 47.5% in OPERA II. Overall, both trials met their primary endpoints and several secondary endpoints, showing a superiority in efficacy of OCR over IFN-β1a [29].

Additionally, in a systematic review including 46 studies, OCR was suggested to have a superior (or comparable) effect over all approved disease-modifying therapies (DMTs) for patients with RMS and a similar safety profile [41].

Kappos et al. [2] performed interesting analyses from the pooled data set of the OPERA I and II studies. They questioned whether disability accumulation in RMS was associated with relapse activity or if underlying progression was the driver for disability accumulation. They found that most disability accumulation in RMS was linked to PIRA and only around 5% of CDA could be explained by RAW, while 4–5% of the patients experienced both RAW and PIRA events at 12-week and 24-week composite CDA consisting of overall (EDSS), upper (9-hole peg test), and lower (25-foot walk) extremity function. OCR appeared to be superior to IFNβ in suppression of both PIRA and RAW.

Results from the extension phase of the phase III trials OPERA I and II have recently been published showing that early and continuous treatment with OCR is clinically as well as radiologically beneficial to patients with RMS with no additional safety concern. Patients who switched from IFN-β1a treatment to OCR experienced a decrease of relapse rates and a near-complete suppression of MRI activity. However, the extent of diminishment of disease activity, measured in this study as CDP, was higher in patients who were treated with OCR for 5 years than those who received IFN-β1a for 2 years before switching to OCR for 3 further years (*p* = 0.014). Hauser et al. [40] therefore concluded that early high-efficacy treatment is a better strategy than traditional escalation approaches.

### OCR in PPMS

Due to hints of efficacy in the subgroup analysis of the OLYMPUS study, a phase III, randomized, placebo-controlled, manufacturer-sponsored study of OCR was conducted in patients with PPMS (ORATORIO). In ORATORIO, 732 patients with PPMS (average age 44.6 years, mean EDSS 4.7, mean disease duration 6.5 years) were randomly allocated to receive either 600 mg of i.v. OCR every 24 weeks (*n* = 488) or placebo (*n* = 244) with a treatment duration of at least 120 weeks. The trial met its primary endpoint, defined as CDP-12 (“percentage of patients with disability progression confirmed at 12 weeks in a time-to-event analysis”) as only 29.6% of the patients in the OCR arm showed disease progression in comparison to 35.7% in the placebo arm. Furthermore, four out of five secondary endpoints were reached: CDP-24 was significantly lower with OCR than placebo (29.6% vs 35.7%), and results of the 25-foot timed walk test favored OCR (29.4% less in OCR group) at week 120. The total volume of brain lesions on a T_2_-weighted MRI was diminished by 3.4% with OCR, while it was increased by 7.4% with placebo. Patients treated with OCR also showed less brain volume loss. Despite these ground-breaking results, the Physical Component Summary score of a standardized 36-item based short-form health survey did not differ significantly [42]. The ENCORE study, which evaluated the effect of OCR on upper limb function in the ORATORIO cohort, showed positive results [43].

The benefits from OCR treatment were confirmed in the long-term follow-up of the ORATORIO trial, with 451 ongoing patients. RMS subjects, who initially received OCR, showed a lower risk of progression on disability measures than those who started off with placebo and only switched to OCR after 120 weeks (EDSS difference of 13.1%, 9-hole peg test difference of 12.5%, timed 25-foot walk difference of 7.5%, composite progression difference of 10.1%, and confirmed time to requiring a wheelchair 7.4% of difference). Furthermore, the percentage change from baseline regarding T_2_ lesion volume and T_1_ hypointense lesion volume was lower in patients with early and continuous OCR treatment. This indicates that early treatment is also beneficial in PPMS [44].

Interestingly, the results are less convincing in comparison to studies evaluating the effect of OCR treatment in RMS, as many of the study participants still showed active enhancing MRI lesions. This leads to the assumption that OCR is beneficial to a certain extent for patients with PPMS; however, it does not fully prevent clinical progression and neurodegeneration. The discrepancy between the results of ORATORIO in comparison to OLYMPUS is probably due to the difference in patients’ characteristics of the two studies. While the mean age of OLYMPUS was 6.1 years higher, the disease duration 2.3 years longer, and the EDSS at enrolment of the study 0.5 higher, the question remains unclear whether OCR also shows beneficial effects for patients outside the inclusion criteria of the study.

In a prespecified subgroup analysis of the ORATORIO study, comparing subjects with and without T_1_ GEL at baseline, a trend for greater treatment effect of OCR was seen in patients with baseline T1 GELs vs without lesions concerning 12-week and 24-week CDP and T2 lesion volume. Furthermore, age subgroup analysis showed a greater magnitude of OCR treatment effect in patients aged ≤ 45 years vs > 45 years regarding 12-week and 24-week CDP, T2 lesion volume and total brain volume change. Importantly, when pointing towards age subgroup analysis, the inverse correlation between age and acute MRI activity in this study group should be kept in mind. Although the study was not powered to subgroup analyses, the data indicate a greater benefit of OCR treatment in younger patients with a more active phenotype of PPMS [45]. These observations are in line with the abovementioned results of the subgroup analysis of younger PPMS patients treated with RTX [28]. Moreover, prespecified subgroup analyses revealed greater effects of OCR treatment in male than in female patients. A trend favoring males was observed regarding 12-week composite CDP (consisting of CDP, the timed 25-foot walk test, and 9 hole peg test), whereas no differences on the basis of sex were detected regarding the annualized relapse rates [45].

In 2017, OCR became the first drug with a proven effect in patients with PPMS and hence represents a breakthrough for these patients.

### Ongoing Trials

With the approval of OCR, several other phase III studies have been initiated. Topics of interests of ongoing trials are the safety profile (NCT03085810, NCT04478591, NCT04387110, NCT03599245) and mode of application of OCR (NCT03972306), its efficiency in different study groups, namely children (NCT04075266) and different ethnogeographical groups (NCT04458688, NCT03784547), its impact on the well-being of patients (NCT03025269, NCT03562975, NCT04448977) and its effect on the immune system (NCT03138525, NCT04459988, NCT02545868, NCT04261790) as well as resident cells from the central nervous system such as microglia (NCT03691077, NCT04230174). Furthermore, as it still remains unclear how OCR actually mediates its clinical benefit, some studies focus on elucidating the mechanism of action (NCT04459988, NCT03344094, NCT02688985) and the reason of interindividual differences of the effectiveness of OCR treatment (NCT03873389, NCT03344094, NCT03523858, NCT01194570, NCT02861014). Therefore, some studies also aim to identify biomarkers to predict clinical benefit of anti-CD20 biologicals (NCT04466150, NCT03396822, NCT04377555). Other important issues in ongoing trials are the comparison of OCR to other CD20 depleting antibodies as well as the switch in therapy from other disease modifying drugs to OCR (NCT03157830, NCT02980042) and its potential interaction with other drugs (NCT04175834).

## Ofatumumab

OFA is a fully human IgG1 mab binding both the large and small CD20 extracellular loop with a high binding affinity and slow off-rate [46]. It was first developed by Genmab and approved by the FDA under the name Arzerra for refractory CLL in October 2009, however in significantly higher doses [47]. Novartis gained rights over the drug (now called Kesimpta) in 2015. OFA targets a different epitope of the CD20 protein than RTX or OCR and results in a stronger CDC than ADCC [26]. After applying the initial doses under medical supervision, OFA can be administered s.c. outside infusion centers (once monthly) and hence represents the first self-administered B cell–depleting drug. This flexibility and autonomy denote a huge benefit for MS patients.

### Preclinical and Phase II Studies

In preclinical studies, it could be shown that s.c. administered antibodies drain through transcytosis in the lymph nodes (LNs) within seconds [48]. Furthermore, in a study performed in cynomolgus monkeys, the effects of subcutaneously administered OFA at human equivalent therapeutic dose were evaluated. Theil and colleagues [49] found that low-dose OFA efficiently and rapidly depletes B cells and CD20^+^ T cells. However, marginal zone B cells in the spleen and LN are spared by the treatment. Further research needs to be conducted to better understand the differential effects of s.c. administered drug in contrast to i.v. administered treatment regarding efficiency, antibody concentration in blood, lymphoid tissue, and brain.

In a phase II trial, published in 2014, 38 RMS patients received different doses of two i.v. infusions of OFA (100 mg, 300 mg, and 700 mg) administered every second week. Compared to placebo, the development of new brain MRI lesions was suppressed with all doses to a similar extent as observed within the OCR trials (> 99% at week 24) [50].

These results, together with the observation of phase I/II trials in patients with RA, where s.c. OFA showed a more favorable safety profile [51], paved the way for a larger dose-finding, double-blind phase IIb trial (MIRROR**)** of subcutaneous OFA. In this study, 232 patients with RMS were randomly allocated to receive either 3, 30, or 60 mg of OFA every 12 weeks, 60 mg of OFA every 4 weeks, or placebo for a period of 24 weeks. With a reduction of at least 65% on new T1 GEL for each dose of OFA at week 12 and even ≥ 90% of reduction in all groups receiving ≥ 30 mg OFA, this study succeeded to meet its primary endpoint. Interestingly, this study showed that a complete B cell depletion was not necessary for a sufficient suppression of relapse activity.

In a second phase II trial (APLIOS), the injection of OFA via a prefilled syringe (PFS) was tested against the use of an autoinjector (AI) pen in patients with RMS. Patients were randomized according the localization (abdomen or thigh) and injection device. B cell counts were determined nine times over 12 weeks and analyzed by measuring the area under the curve (AUC) from start to end of a 4-week dosing interval. Across all groups, OFA was found to effectively decrease B cell counts to bioequivalent levels. Therefore, the trial concluded that the patient-friendly AI pen can be used for monthly at home administration [52].

### Phase III

Due to these remarkable results, two identical double-blinded, double dummy, phase III trials (ASCLEPIOS I and II) were conducted in patients with RRMS or SPMS. The aim was to compare the efficacy and safety of OFA and teriflunomide (TERI) over 30 months. In total, 1882 patients were included in the studies. Nine hundred forty-six patients received 20 mg of OFA s.c. every 4 weeks (after 20 mg loading doses at days 1 and 7) and 936 patients received 14 mg/day of TERI orally. Patients were aged between 18 and 55 years (mean age of 38 years), with a mean EDSS of 2.9 and an average disease duration of 8 years. Both trials hit their primary endpoint: The annualized relapse rate was 0.11 (ASCLEPIOS I) and 0.10 (ASCLEPIOS II) with OFA and 0.22 (ASCLEPIOS I) and 0.25 (ASCLEPIOS II) with TERI representing a relative reduction of 50.5% and 58.5% (*p* < 0.001). Already after 3 months of treatment, significant effects were seen in pooled analysis: The risk of disability worsening at 3 and 6 months was significantly lower with OFA. Furthermore, OFA reduced the total number of T1 lesions in MRI by 97.5% (ASCPLEPIOS I) and 93.8% (ASCLEPIOS II). The neurofilament light concentration in the serum of OFA-treated patients was also significantly reduced compared to the comparison arm. The change in brain volume loss did not change significantly compared to TERI. Hauser et al. [31] do not provide a possible explanation for this discrepancy between the two brain markers. However, one has to keep in mind that TERI itself prevents brain volume loss, which might be an explanation for OCR not showing any superior effect regarding this parameter if compared to TERI [53, 54]. Interestingly, when regarding discontinuation of treatment, the percentage of patients who terminated the application of the drug was higher in the TERI group than with OFA (21.2% vs 14%) [31].

A subgroup analysis of the ASCLEPIOS trials evaluated the benefit-risk profile of OFA in treatment-naïve early RRMS patients, who represented about one-third of the patients in the study. It showed that OFA reduced the annualized relapse rate by 50.3% vs TERI. Furthermore, the 3-month confirmed disability worsening (CDW) was reduced by 38%, while the 6-month CDW risk was reduced by 46% with OFA. Moreover, OFA significantly reduced gadolinium-positive T_1_ and T_2_ lesions. The odds of achieving NEDA-3 (defined as absence of relapses, disability worsening, and MRI activity) was 3-fold higher at the first year and even 14-fold higher at the second year of treatment [55]. These data emphazise that early high efficacy treatment is necessary in order to achieve the best possible long-term outcome for MS patients [56].

In August 2020, the FDA approved the subcutaneous use of OFA as treatment for RRMS, SPMS, and CIS. Thus, OFA is the first and only approved self-administered B cell depleting therapy for MS.

A recently published meta-analysis, comparing OFA and other disease-modifying therapies for relapsing multiple sclerosis, concludes that OFA is as effective as other highly efficient DMT such as alemtuzumab, natalizumab (NAT), and OCR [57].

### Ongoing Trials

As OFA has only recently been approved, the number of clinical studies is still limited. Up to now, one ongoing phase III clinical study evaluates the safety, tolerability, and efficiency of OFA in patients with RMS (NCT03650114). An additional ongoing study evaluates, similar to studies on OCR, the effects of OFA on cells of the CNS, specifically microglia (NCT04510220). Furthermore, two other studies elucidate the effectiveness and advantages in patients switching from DMTs, namely dimethylfumarate or fingolimod, to OFA (NCT04353492) as well as in patients switching from other anti-CD20 therapy to OFA (NCT04486716).

## Ublituximab

UB, first introduced by TG therapeutics, represents a novel, glycoengineered anti-CD20 antibody. It is currently studied in patients with chronic lymphocytic leukemia (CLL) and has gained Fast Track Designation by the FDA for this indication [58]. Its effect is mainly mediated through ADCC. Moreover, it is supposed to allow lower doses and shorter infusion times in comparison to other anti-CD20 biologicals [59, 60]. Until now, there is only a limited number of trials published that studied the clinical benefit of UB in patients with RMS.

### Phase II

In a phase II placebo-controlled study, different doses and infusion times of UB were tested in patients with RMS. Forty-eight RMS patients with a mean age of 39.2 years, a mean EDSS of 2.44, and a mean disease duration of 7.4 years received in total three UB infusions. After an initial infusion of 150 mg UB over 1–4 h, patients received a second and third infusion with 450 to 600 mg UB over 1–3 h after 14 days and at week 24. The follow-up of the study was in total 48 weeks, and the primary endpoint was defined as the level of B cell depletion. Under UB treatment, 99% of circulating B cells were depleted by week 4 and stayed at low levels at weeks 24 and 48 [61]. Furthermore, a shift towards more naïve and regulatory T cells was detected [62]. The number of T_1_ GEL was reduced from the baseline being 3.63 to 0 at week 24 (*p* = 0.003) and was maintained at week 48 (*p* = 0.016). Furthermore, a total decrease of 10.6% was observed in T_2_ lesion volume (*p* = 0.002). Ninety-three percent of patients treated with UB were relapse free at week 24, 74% of patients fulfilled NEDA criteria, and the annualized relapse rate was 0.07. Due to these encouraging results, UB is suggested to be a safe and efficient drug with a rapid infusion time of 1 h [61].

### Phase III/Ongoing Studies

Two identical but independent double-blinded, multicenter, placebo-controlled phase III trials (ULTIMATE I and II) have been launched to evaluate the effect of 450 mg of UB i.v. in comparison to TERI (NCT03277261, NCT03277248). The primary endpoint of these studies is defined as annualized relapse rate after 96 weeks of treatment. Completion dates of these studies are September 2021. Furthermore, in November 2019, an open-label, single-arm extension study of these studies was launched to study the long-term efficiency and safety profile of UB in subjects with RMS (NCT04130997). Results from the open-label extension study are expected for 2023. Moreover, an extension phase of the phase II trial is run and will reach study completion in December 2021 (NCT03381170).

## Comparison of CD20 Antibodies—the Pros and Cons

### Pharmacological Characteristics

With the approval of OFA by the FDA in August 2020, two different anti-CD20 depleting antibodies, namely OCR and OFA, are officially available for the treatment of MS. RTX is not approved for MS; however, it is still used as an off-label therapy. As the anti-CD20 antibodies possess slightly different properties regarding their mechanism of action, immunogenicity, adverse effects, and administration form, it is of interest to find out which antibody is suitable for which patients. Furthermore, the aim of current research is to develop new antibodies with improved properties and greater clinical effects. Therefore, it is important to obtain a better understanding of the pharmacological differences in these antibodies. In the following section, the mechanistic differences between RTX, OCR, OFA, and UB will be compared and highlighted.

#### Mode of Action

IgG anti-CD20 antibodies consist of two light and two heavy chains and can be grouped in type I and type II antibodies regarding their mechanism of action [26]. Until now, all anti-CD20 mabs studied in trials with patients diagnosed with MS belong to the group of type I antibodies. The mechanisms of action of type I antibodies are based on ADCC or antibody-dependent cellular phagocytosis (ADCP) and CDC rather than on non-apoptotic form of programmed cell death (PCD) and ADCC [26] (see Table 1). Type I mabs crosslink two CD20 tetrameters and have the ability to translocate CD20 into large lipid microdomains (called lipid rafts) within the plasma membrane. As a result, recruitment and activation of the complement system is a strong mode of action [38]. As OFA is binding the large and small extracellular loop, it has been hypothesized that OFA is closer to the cell surface than other anti-CD20 mabs and therefore results in the strongest CDC so far within the anti-CD20 group [63, 64]. This may be of specific interest when considering key effector mechanisms in the CNS where the complement system plays an important role in immunity [65, 66]. Furthermore, OFA is thought to dissolve more slowly from the CD20 antigen, also resulting in a stronger efficacy [67]. Another interesting feature of OFA, in contrast to RTX, is the maintenance of high CDC in the presence of low CD20 expression [64].

**Table 1:** Overview of the relevant anti-CD20 in MS therapy and their mode of action

Antibody (chronological order)	Type	Mode of action		Dose and application mode	Clinical trials	Special features
		ADCC	CDC			
Rituximab	Chimeric IgG1	+ +	+ +	1000 mg, no approved protocol	RRMS HERMES phase II PPMS OLYMPUS phase III	Prototype CD20 antibody
Ocrelizumab	Humanized IgG1	+ + +	+	600 mg i.v., every 6 months	RRMS phase II RRMS OPERA I + II phase III	First approved drug for the treatment of PPMS
Ofatumumab	Fully human IgG1	+ +	+ + +	20 mg s.c., monthly	RRMS phase II RRMS MIRROR phase II RRMS ASCLEPIOS I + II phase III	First approved self-administered B cell–depleting therapy
Ublituximab	Chimeric IgG1 with low fucose content	+ + + +	+	450 mg i.v. (1 h), every 12 weeks	RRMS phase II RRMS ULTIMATE I + II phase III	High affinity to FcγRIIIa due to low fucose content

ADCC activity is dependent on the fucose content of the oligosaccharides bound to the anti-CD20 mab. As a result, antibodies that have a low fucose content perform higher ADCC activity [68]. The potency of this mode of action is related to the individual affinity of an antibody to the Fc receptor FcγRIIIa, expressed on a range of effector cells such as monocytes and natural killer (NK) cells. The different affinities to this specific Fc receptor as well as the different binding epitopes result in slight differences between the second-generation anti-CD20 mabs and its precursor RTX [38]. UB was manufactured with a low fucose content in order to enhance ADCC/ADCP. This enhancement is facilitated through a strong affinity of the antibody to the FcγRIIIa and results in a high NK cell-mediated ADCC regardless of CD20 surface expression [67]. UB possesses the highest efficacy regarding ADCC within the current anti-CD20 mabs [60]. Similar to UB, the mode of action of OCR also concentrates on ADCC and less on CDC resulting in a, most likely, more efficient mode of action regarding tissue-dependent pathogenic mechanisms than RTX and OFA [39].

#### Immunogenicity

Due to their fully humanized or human structure, OCR and OFA are considered to be less immunogenic than their chimeric predecessor RTX and the currently tested UB. Consequently, the probability of anaphylactic reaction should be minimized [69]. The production of antidrug-binding antibodies is seen as an indicator for immunogenicity. In the HERMES and OLYMPUS trials, the incidence of human antichimeric antibodies (HACA) in the RTX group was 24.6 and 7% [27, 28]. In contrast, the pivotal studies on OCR and OFA showed a much lower incidence of antidrug-binding antibodies in the OCR and OFA group (OPERA I and II 0.4%; ORATORIO 1.9%; ASCLEPIOS I and II 0.2%) [29, 31, 42]. In a phase II trial on intravenous use of OFA in RRMS, none of the patients developed human antihuman antibodies (HAHA) [50]. None of the abovementioned trials on RTX showed a positive association between positivity for antidrug-binding antibodies and the type of adverse events or the efficacy of the response to the treatment described [27, 28]. Regarding UB, it still needs to be determined whether the chimeric antibody has a heightened immunogenicity.

In 2005, a case report was published describing a patient suffering from severe systemic lupus erythematosus (SLE) who developed tolerance to RTX. The first two RTX infusions led to sufficient B cell depletion and were followed by clinical improvement. When the patient received two further RTX infusions 1 year later, she responded neither serologically nor clinically, but developed a HACA response. Years later, after other therapies failed in controlling the progression of the disease, another anti-CD20 antibody, namely hA20 (veltuzumab), was applied. The humanized antibody led to a B cell depletion and a clinical improvement [70]. On the one hand, this report emphasizes the importance of developing antibodies that are not or little immunogenic in order to avoid resistance to drugs. On the other hand, it demonstrates the possibility of switching to another anti-CD20 antibody and reobtaining clinical efficacy.

Recently, a register study on adverse events (AEs) associated with RTX or OCR treatment of MS patients reported in the Food and Drug Administration’s Adverse Event Reporting System was published. This study demonstrated a two times higher proportion of AEs concerning the immune system in patients treated with RTX. Since the category of AEs concerning the immune system included hypersensitivity and anaphylactic shock, the higher proportions of AEs within the RTX associated reports might indicate more severe allergic reactions to the RTX infusion due to a higher immunogenicity. This observation goes in line with the incidence of antidrug antibodies in the clinical trials shown above. Furthermore, a higher rate of reported infections associated to OCR was observed in this study. However, in their analysis, the number of patients treated with OCR or RTX with no AE was not registered, so that final conclusions are difficult to draw. Here, a head-to-head study would give more precise and reliable data [71].

### Safety Profile

RTX, OCR, OFA, and UB are overall well-tolerated drugs. This is probably due to the fact that OCR as well as other anti-CD20 treatments target most efficiently circulating B cells. As only 1/50 of all B cells is present in the peripheral blood, the survival of tissue-residing B cells might be crucial for the relatively small number of reverse effects. [72].

**Infusion or injection-related reactions (IRRs)** are common adverse events in the clinical studies on anti-CD20 antibodies in MS and include among others nausea, pruritus, chills, rash, throat irritation, and flushing. In studies, IRRs were more common in the anti-CD20 group as compared to placebo and appeared mostly during the first infusion/injection, decreasing with subsequent administrations [27–29, 40, 42, 61].

In the HERMES trial, the incidence of IRRs within 24 h after the first infusion was 78.3% in the RTX group and 40.0% in the placebo group. After the second infusion, the incidence in the RTX group declined to 20.3% while the incidence in the placebo group remained at 40.0%. Most of the reported IRRs in the RTX group were mild or moderate (grade 1 or 2 according to the Common Terminology Criteria for Adverse Events by the National Cancer Institute), while only 7.4% were categorized as grade 3 events. No grade 4 events occurred. No premedication with glucocorticoids prior to RTX infusions took place to prevent IRRs. Patients in the HERMES trial experienced among other IRRs fever, chills, rigors, nausea, pruritus, asthenia, and hypotension—symptoms that were also associated with cytokine release syndrome during B cell lysis in RTX-treated patients with chronic lymphocytic leukemia (CLL) [27, 73]. In the OLYMPUS trial, the frequency of IRRs associated with the first infusion was higher in the RTX group compared to placebo (67.1% vs 23.1%), containing primarily mild to moderate AEs. By week 74, the percentage of IRRs decreased to lower levels than in the placebo arm (4.9% vs 7.2%) [28].

In the OPERA and ORATORIO trials on OCR, all patients received one 100 mg dose of i.v. methylprednisolone prior to each infusion to prevent IRRs. Prophylaxis with antihistamine and an analgetic or antipyretic was recommended but not obligatory. In both trials, more patients experienced at least one IRR in the OCR group compared to the IFN-β1a and placebo arm. IRRs decreased in frequency and severity over the administration of subsequent doses, as previously observed in RTX studies [29, 42]. In the OPERA studies, most of the IRRs were mild to moderate, but one OCR receiving patient experienced a life-threatening episode of bronchospasm during the first infusion and was withdrawn from the trial [29]. Regarding ORATORIO, no fatal or life-threatening IRR occurred. Nevertheless, two patients in the OCR (0.4%) withdrew from the treatment due to IRRs [42].

Patients in the MIRROR and ASCLEPIOS trials on OFA also showed higher frequencies of IRRs in the anti-CD20 arm in comparison to the placebo or TERI arm, mostly occurring in association with the first injection (MIRROR 41–66% vs 15%; ASCLEPIOS 20.2% vs 15.0%). Most IRRs in both studies were classified as mild or moderate, but two participants of the ASCLEPIOS studies receiving OFA experienced grade 3 injection-related systemic reactions (0.2%) [31, 74]. In the MIRROR study, acetaminophen and an antihistamine were administered orally up to 2 h before each injection to prevent AEs [74], whereas in the ASCLEPIOS trials, premedication was optional. Less than 70% of the subjects used premedication (steroids, acetaminophen, or antihistamines) for the first injection, with decreasing usage before subsequent administrations. The comparison of the incidence of IRRs with or without premedication did not show a conclusive benefit of premedication [31].

In a phase II placebo-controlled trial on UB, the incidence of IRR within the UB group was 50%. In this trial, all patients received an oral antihistamine, an oral corticosteroid before the UB infusion, and the same initial dose of UB. For the following infusion, higher doses and faster infusion times where tested. Fox et al. [61] concluded that higher doses and faster infusion times do not correlate with higher rates of IRRs.

Studies evaluating the side effects of RTX in patients with low-grade non-Hodgkin’s lymphoma showed that infusion reactions during RTX treatment are most likely associated to the complement activation [75]. Based on this knowledge and the potentially higher immunogenicity of the chimeric RTX, it is assumed that RTX has a higher risk of infusion reactions than OCR. One possible reason is that OCR, in contrast to RTX, is known to mediate its effect more through ADCC than CDC. However, due to inconsistent premedication, the incidence of IRRs in the different pivotal studies cannot be compared. In order to evaluate the safety of RTX, OCR, and OFA, as well as other anti-CD20 treatments like UB, a head-to-head randomized controlled trial would be the gold standard.

To decrease the risk of IRRs, a premedication with 100 mg of i.v. methylprednisolone (or an equivalent corticosteroid) 30 min prior to the OCR infusion is recommended in the prescribing information. Moreover, an antihistamine should be administered approximately 30 to 60 min before each infusion. Additionally, an antipyretic can be considered [76]. Due to the limited effect of premedication on IRRs in the ASCLEPIOS studies, the prescribing information does not include any recommendation for the use of premedication prior to OFA injections [77].

**Infections** are common AEs associated with the use of drugs that are modulating the immune system. Therefore, they should be considered when examining the safety profile of anti-CD20 drugs.

In the two phase II trials on RTX in MS, the incidence of any infection was similar in the RTX group and placebo (HERMES 69.6% vs 71.4%; OLYMPUS 65.3% vs 68.2%). Both trials reported no significant risk of opportunistic infections [27, 28]. In the HERMES trial, the percentage of infection-associated serious AEs was even lower in the RTX group in comparison to placebo (2.9% vs 5.7%) [27].

Regarding OCR, in the OPERA and ORATORIO trials. the infection rates in the OCR receiving groups were also similar to the comparison arm (OPERA I 56.9% vs 54.3%; OPERA II 60.2% vs 52.3%; ORATORIO 71.4% vs 69.9%). The most common infections acquired by OCR receiving patients were nasopharyngitis, upper respiratory tract infections, urinary tract infections, and influenza [29, 42]. In the OPERA trials, the incidence of serious infections was, just like in the HERMES trial, lower in the anti-CD20 group than in the comparison arm (1.3% vs 2.9%). Herpesvirus-associated infections were more common in the OCR receiving patients than in the IFN-β1a cohort. However, all of them—except for one case of severe genital herpes—were of mild to moderate severity [29]. In the ORATORIO trial, the percentage of patients experiencing serious infections was similar in both groups (OCR 6.2% vs placebo 5.9%), but the incidence of herpesvirus-related infections was higher in the OCR group compared to placebo (4.7% vs 3.3%) [42]. One potential serious opportunistic infection was reported during the OLE of the ORATORIO study. One patient who received cancer chemotherapy experienced a serious candida sepsis 11 months after cessation of OCR treatment [44].

In the phase II MIRROR trial and the ASCLEPIOS studies on OFA, the overall rates of infection-related AEs in the OFA group were also similar to those of the comparison arm (MIRROR OFA 27% vs placebo 25%; ASCLEPIOS 51.6% vs 52.7%). In none of the trials, opportunistic infections were reported [31, 74]. The most common infections were similar to those observed in the pivotal trials on OCR. OFA-receiving patients of the ASCLEPIOS trials experienced more serious infections than patients receiving TERI (2.5% vs 1.8%), whereas they only differed marginally in the incidence of herpes infections (OFA 4.9% vs 4.2% TERI) [31]. Interestingly, an imbalance in the incidence of appendicitis was observed. The percentage of patients suffering from an appendicitis was almost four times higher in the OFA group than placebo (8 of 946 vs 2 of 936). However, neither in phase II studies nor in studies on other anti-CD20 therapies a higher risk for appendicitis was observed [27, 29, 31, 50, 74].

The phase II trial on UB in RMS patients reported no serious infections. The profile of documented infections was similar to those reported on RTX, OCR, and OFA. Nevertheless, results of the phase III trials ULTIMATE I and II need to be conducted to make a clear statement about the safety profile of UB.

Progressive multifocal leukoencephalopathy (PML) is a feared demyelinating disease of the CNS caused by JC polyoma virus (JCV). In MS treatment, PML is mainly known as a rare but severe complication of NAT treatment [78]. Although no cases of PML have been reported in the abovementioned pivotal clinical trials on RTX, OCR, OFA, and UB, few reports indicate an association of anti-CD20 treatment and an increased risk of developing PML. Carson et al. [79] recapitulated 57 cases of PML development after RTX treatment including 52 patients with lymphoid malignancies, two patients with SLE and one subject treated for RA, idiopathic autoimmune pancytopenia and immune thrombocytopenia purpura. All patients were HIV-negative. The causal assessment is difficult because all patients received other additional drugs that affect the immune function (for example, corticosteroids or alkylating agents) and PML has also been reported among patients with lymphomas who did not receive RTX. In 2011, a report on four cases of PML in RTX-treated patients with RA was published. All four patients received other immune-modulating drugs prior to the RTX infusions while two of them also received chemotherapy due to malignancies [80]. The same group presented data of MS patients who were diagnosed with PML during OCR treatment in 2019. All of the seven confirmed cases of PML were classified as carryover PML as the disease was only detected a few months after switching from a PML-associated DTM—namely NAT and fingolimod—to OCR. Six patients had received NAT prior to the OCR infusions, and one patient had received fingolimod [81]. Another study collecting data of 42 patients who switched from NAT to OCR showed contrary results. None of the patients, who switched from NAT to OCR due to a JCV index of > 1.5 and either a long duration of NAT treatment or their personal wish to avoid PML risk associated to NAT, developed PML. This observation strongly supports the idea of switching from NAT to OCR in case of a high risk of PML [82]. These findings are emphasized by a retrospective study on patients from three centers in Sweden who switched from NAT to other DMTs including RTX due to JCV positivity and even showed favorable effects of RTX on the suppression of clinical relapses, adverse events, and treatment discontinuation with a decreased risk for PML [83]. Furthermore, a recent report that determined anti-JCV seropositivity and anti-JCV antibody index (AI) in patients before and after the initiation of OCR described no significant change in anti-JCV AI and reported only a single seroconversion in 20 initial seronegative patients [84].

Although OLE studies on OCR over a study period of 5 and 6.5 years did not report any cases of PML, awareness of a possibly increased risk of developing PML under anti-CD20 therapy is still needed [40, 44].

**Hypogammaglobulineamia** was described as a result of long-term RTX therapy in patients with other autoimmune diseases and lymphoma patients (reviewed by Sacco et al. [85]). Hypogammaglobulineamia represents a major safety issue of any drug. As it is especially of interest for long-term treatment, it is important to evaluate the Ig levels of patients with MS receiving anti-CD20 treatment. Regarding RTX, at week 72 of a phase I, trial IgG or IgA levels of all RTX receiving patients were not lower than the lower limit of normal (LLN), but 44% of the patients with normal baseline IgM levels presented values below the LLN at week 72. This subgroup of patients had a higher incidence of overall infections but did not experience any severe or serious infections [22]. The proportion of patients with IgM levels below the LLN was also significantly higher within the RTX-receiving groups of the two phase II trials HERMES and OLYMPUS than the placebo arms (HERMES 22.4% vs 3.0%; OLYMPUS 31.7% vs 5.9%) [27, 28]. During the OLE phase of the OPERA studies, 5.4% of the OCR receiving patients had IgG levels below the LNN at year 5, while the percentage of patients with levels below the LLN was 5.4% for IgA and 29.5% for IgM [40]. Data acquired within the OLE phase of the ORATORIO study revealed a decrease of IgG and IgA below the LLN in 5% of the OCR-treated patients, while the incidence for a decrease of IgM below the LLN was 29% at study year 6.5 [44]. Both trials OPERA and ORATORIO showed an overall decline of IgG levels, even though the mean IgG levels stayed above the LNN [86]. Recently presented data of a 120-week observation of patients receiving OFA as part of the ASCLEPIOS studies showed an overall stable level of IgG. Interestingly, the IgM levels were reduced by week 120, even though the mean IgM levels were still above the LNN [87]. More long-term safety data are awaited to see whether the trend of IgM levels might lead to a reduction below LLN. Furthermore, it needs to be determined whether the less deep depletion than that caused by OCR keeps IgG levels stable during longer treatment periods. Since anti-CD20 depletion does not target plasma cells directly, a possible reduction of Igs has not been observed directly after initiation of the treatment and more long-time assessments are required.

#### Malignancies

Anti-CD20 antibodies lead to an immunosuppression, which can result in an impaired immune surveillance of newly degenerated cells. Therefore, the incidence of new neoplasms should be analyzed in order to detect a possibly increased risk of malignancies.

The ORATORIO trail on PPMS showed an imbalance in the incidence of new malignancies between the OCR group and the placebo group. Of the patients receiving OCR, 2.3% were newly diagnosed with a malignancy, while the incidence in the placebo group was 0.8%. Of 488 OCR-treated patients, four women developed breast cancer, three patients were newly diagnosed with basal cell carcinoma, and one patient was diagnosed with anaplastic large cell lymphoma, endometrial adenocarcinoma, malignant fibrous histiocytoma, and metastatic pancreatic carcinoma, respectively. In an OLE phase, in which all patients were treated with OCR, two more neoplasms were detected—one basal cell skin carcinoma and one squamous cell carcinoma [42].

In the OPERA trials, 0.5% of the patients receiving OCR were diagnosed with neoplasms (two cases of breast cancer, one case of renal cell carcinoma, and one case of malignant melanoma) while the incidence in the IFN-β1a group was 0.2% (one case of mantle cell lymphoma and one case of squamous cell carcinoma).

Taken together, the data collected from the OPERA trials, ORATORIO, and one phase II study until June 30, 2016, the overall incidence of a first neoplasm among MS patients receiving OCR was 0.4 per 100 patient-years of exposure vs 0.2 per 100 patient-years of exposure in the comparison arm [29, 39, 42]. This rate is within the range of epidemiological data of cancer incidence in MS patients determined by Danish medical registers [88].

In the phase II MIRROR trial on OFA, one case of malignant melanoma was detected during the individualized follow-up of a patient who received 60 mg OFA every 4 weeks [74]. In the ASCLEPIOS trials, the incidence of neoplasms was similar in both groups (0.5% for OFA vs 0.4% for TERI) [31].

Although higher rates of malignancies were reported in some of the pivotal studies for the approved anti-CD20 antibodies, most studies do not support this finding. Pivotal studies on RTX and UB showed no higher incidence of neoplasms in the treatment group than in the comparison arm [27, 28, 61]. The only reported neoplasm during the HERMES trial was a malignant thyroid neoplasm [27]. A Swedish register study investigated the cancer risk of RTX, NAT, and fingolimod compared to each other and to the MS-free general population. The cancer rates within the RTX-treated MS population were similar to the general population [89]. A 9.5-year follow-up of RTX-treated patients with RA does not indicate an increased incidence of malignancies following accumulated exposure with RTX either compared to a placebo group and the incidence in the general US population [90].

In summary, IRRs are the most common AE of anti-CD20 therapy, but their severity is mainly mild or moderate. A final conclusion regarding the occurrence of PML and malignancies cannot be drawn. Nevertheless, the data do not indicate an elevated risk regarding PML or malignancies to this point. The awareness for possible complications, infections, and possible results of decreased Ig levels should remain.

## Perspectives and Outlook

The use of anti-CD20 antibodies in MS therapy has been an important step not only for the treatment of MS but also for the understanding of the disease and its pathology. The stunning results of the phase II clinical trials on RTX already demonstrated that B cell depletion is an efficient way to prevent relapses in RMS and to reduce the development of new inflammatory CNS lesions [27, 28]. For patients with PPMS, the introduction of CD20-depleting therapies in MS treatment was a real milestone, as no disease-modifying drug showing clinical benefits in PPMS patients was available until the approval of OCR in March 2017. This change was long-awaited and even though OCR remains the only therapy for PPMS until now, research is focusing on identifying further biologicals for the approval of PPMS. Generally, CD20-depleting therapy is well tolerated, with infusion reactions and infections being the most common AEs [29]. Moreover, the treatment is relatively easy to monitor because the B cell depletion, and therefore, the therapeutic success can easily be determined through the count of CD19^+^ cells [91]. The effectiveness of anti CD20 antibodies showed that B cells are an appropriate target for MS therapy. These remarkable results provided impetus for further research not only on possible pathogenic function of B cells or certain B cell subtypes but also on the possible regulatory effect of B cells on autoimmunity. Moreover, the beneficial effects of anti-CD20 antibodies triggered interest in exploring other B cell targeting strategies as therapy options, exemplarily Bruton’s tyrosine kinase (BTK) inhibitors.

### Effects of Anti-CD20 Therapies on Immune Cells Other Than B Cells

#### Effect on T Cells

As described above, the beneficial effect of anti-CD20 therapy is thought to be mediated by the depletion of B cells with pathogenic functions. Intriguingly, not only B cells are targeted by anti-CD20 antibodies but also a subpopulation of T cells expressing CD20 on their surface. The existence of CD20^+^ T cells has already been known for almost 30 years [25]. Still, not much attention has been paid to this subtype. CD3^+^CD20^+^ T cells are a heterogenous subpopulation of T cells, including CD4^+^ helper and CD8^+^ cytotoxic subsets [92]. CD20^+^ T cells represent about 2.4% of CD45^+^ lymphocytes and make up around 20% of all CD20^+^ cells [93]. With a higher frequency of CD8^+^ and CD45RO^+^ cells, CD20^+^ T cells possess constitutively and upon activation a more pro-inflammatory profile in healthy donors [94]. In the peripheral blood of patients with RA, CD3^+^CD20^+^ cells have a higher pro-inflammatory capacity than CD3^+^CD20^+^ cells of healthy controls [95]. In untreated MS patients, CD20^+^ T cells were increased compared to healthy controls and remained depleted for at least one year after the last RTX infusion [92]. It could be shown that RTX, OCR, and most likely also other anti-CD20 biologicals do not only target B cells but also achieve a near-complete depletion of CD20^+^ T cells within the first weeks of treatment [92, 93, 96]. Notably, a small amount of CD20^+^ T cells as well as B cells remains in the patients’ blood after application of an anti-CD20 antibody. Taking into account that MS patients contain modestly higher frequencies of CD3^+^CD20^+^ cells, which are predominantly CD8^+^ T cells, it is important to invest more research in this specific subtype in order to study their potential role in the pathogenesis of MS. Moreover, the question arises whether the clinical benefit of anti-CD20 therapies in MS is partly based on the depletion of these CD20^+^ T cells.

#### Effect on myeloid APCs

Interestingly, B cell depletion seems to enhance pro-inflammatory properties of monocytes. In experiments studying the effects of anti-CD20 depletion on monocytes, a B cell–independent EAE model was used in which mice were immunized with MOG peptide followed by the depletion of T_regs_ and CD20-positive cells. Monocytes themselves do not express anti-CD20, but the absence of B cells alters the activation status of monocytes. Regulatory T cells were depleted prior to the induction of EAE using anti-CD25 to eliminate their effect on monocytes. The depletion of T_regs_ itself slightly worsened the disease, while the additional anti-CD20 injection led to an exacerbation of EAE. When characterizing the CD11b^+ ^cells of these B- and T_reg_-depleted mice, Lehmann-Horn et al. [97] found that they produced significantly higher levels of TNFα in comparison to monocytes of untreated controls. In order to validate the results found in EAE, a human ex vivo study was conducted. Patients included in this study were diagnosed either with MS, neuromyelitis optica (NMO), myasthenia gravis, or autoimmune neuropathy. As a parameter for monocyte activation, TNFα production and the expression of the costimulatory activation marker signaling lymphocytic activation molecule (SLAM) were determined. When reactivated by 250 pg/ml LPS ex vivo, monocytes of B cell–depleted patients produced higher amounts of TNFα than monocystes of untreated controls. Furthermore, the treatment group contained a higher proportion of samples in which monocytes showed an overall higher expression of SLAM. The samples of the group receiving RTX showed a wider range of values than the control group in TNFα production and SLAM expression. To conclude, the more pro-inflammatory state of myeloid APCs can be explained by the depletion of IL10-producing B cells with a regulatory function due to RTX treatment since IL10 regulates the function of monocytes [98].

### Repletion of CD20^+^ Cells

CD20-depleting therapies show outstanding effects on relapse biology, but they also display immunological changes and can alter the immune system. The question whether this modification of the immune system is temporary or persistent is of great interest as B cells might repopulate in a transformed way leading on the one hand to new safety issues and on the other hand to different dosing strategies. The analysis of the immune repertoire prior, during, and after cessation of anti-CD20 therapy is of utmost importance to characterize repopulation kinetics and possible long-term alterations in the B cell pool.

As CD20 is not expressed on all types of B cells, precursors of B cells survive in blood and lymphoid tissues resulting in a time-delated replenishment of the B cell pool when ceasing the treatment. So far, repopulation kinetics and depth of depletion are dose and frequency dependent [74, 91]. Whether the duration of B cell–depleting treatment regimens also plays a role in repopulation characteristics still needs to be clarified.

In a study, in which RMS patients were treated with RTX, B cells started to repopulate 25 to 36 weeks after the last RTX infusion [92]. In a phase I trial, B cells were found to reach 34.5% of the baseline level at week 72 post-RTX infusion with the majority being naïve B cells [22]. Similar results were seen in the phase II trial, in which 30% of baseline values were reached 48 weeks post-treatment [27]. Regarding OCR, the time to repletion of B cells to baseline level was reached at a median of 62 and 72 weeks after three and four OCR cycles in phase II studies [99]. In the MIRROR study, evaluating different doses of s.c. OFA and different frequencies of OFA administration not only a dose-dependent B cell depletion but also repletion could be observed. The median time to near complete repletion was around 11 months for patients who received either 3 or 30 mg of OFA every 12 weeks and 14 months for subjects receiving 60 mg of OFA every 4 or 12 weeks. Interestingly, B cells started to repopulate between the administrations when OFA was applied every 12 weeks. In contrast, B cell levels were kept low in patients who received OFA more frequently (every 4 weeks). Furthermore, Bar-Or et al. [74] observed a longer time to replenishment in the higher-dose than lower-dose groups. It is unclear whether different repopulation kinetics is due to real pharmacological differences of the antibodies or whether they are biased by different dosing strategies. Investigations in non-human primates found that post-treatment immune recovery was significantly faster in OFA-treated cynomolgus monkeys (1 mg/kg s.c.) in comparison to RTX-treated primates (10 mg/kg i.v.) [100]. Pivotal studies are pointing towards alterations between the antibodies regarding time of repletion; however, these observations should be treated with caution due to abovementioned reasons. Until now, no head-to-head clinical trial comparing repopulation kinetics of the different anti-CD20 antibodies was performed in humans.

An important observation from pivotal studies is that the clinical benefit of anti-CD20 treatment exceeds the nadir of CD20 positive cells. These findings are especially interesting as some authors attribute lasting effect of anti-CD20 therapy to the absence of CD27^+^ B cells (reviewed by Baker et al. [101]). Memory B cells are suggested to be the trigger of disease activity and the relevant cells to target in order to silence MS pathology. In line with this, Leandro et al. [102] observed that patients with RA tend to relapse earlier with increasing numbers of memory B cells in the reconstitution pool. Currently, CD19 is used as a parameter in order to evaluate repopulation of B cells and the necessity of treatment. However, in case of depletion of memory B cells being the reason for positive treatment effects of anti-CD20 antibodies, a personalized, retreatment depending on the level of circulating CD27^+^ B cells could be considered as a more adequate indicator for treatment necessity. This might allow greater intervals between anti-CD20 injections [101].

Recent research has improved the understanding on immunological characteristics of repleting CD20^+^ cells. A lately published study indicates that the reappearing pool of B cells after depletion with RTX mainly consists of naïve and transitional B cells, while memory B cells are diminished [103]. The same holds true for patients treated with RTX for various autoimmune diseases and for CD20 depletion in EAE induced with MOG peptide [102, 104–106]. Interestingly, in EAE, B cells repopulate after anti-CD20 depletion with a higher amount of memory B cells when using the MOG protein immunization model, which points towards a pathologic reactivation in the presence of auto-antigen [104]. This observation is important when comparing effects of anti-CD20 treatment in MS vs NMO or MOG antibody–associated patients, as in the latter scenario, peripheral activation of regrowing B cells is likely. Since memory B cells are thought to be more pathogenic than naïve B cells, the observation of a more naïve repleting B cell pool seems to be a promising observation. However, repleting naïve B cells in patients with MS and RA were found to possess a more pro-inflammatory phenotype with variations of repopulation kinetics and patterns at an individual level [102–104]. More research is needed to find out whether rebound effects could occur after cessation of long-term anti-CD20 treatment.

A subpopulation of T cells known as CD3^+^CD20^+^ is also efficiently depleted by anti-CD20 mabs since CD20 is to a certain extent also expressed on this subpopulation. With cessation of anti-CD20 treatment, CD20^+^ B cells and also CD3^+^CD20^+^ cells repopulate. Palanichamy et al. [92] observed that CD3^+^CD8^+^CD20^+^ T cells started to repopulate earlier than CD20^+^ B cells (approximately 13–24 weeks after the last infusion of RTX). Schuh et al. [94] confirmed this observation and further detected an inverted ratio of CD20^+^ T to CD20^+^ B cells in the recovery phase. Three of eight investigated MS and NMOSD patients relapsed in the first 8 months after cessation of the treatment and expressed a higher number of CD20^+^ T cells at that time than untreated patients.

In a retrospective study including 45 neurologic patients (mainly with MS or NMOSDs) treated with either i.v. RTX or OCR, a faster reconstitution of B cells was associated with a higher body surface area (BSA). This association persisted even when treatment doses were adjusted to the BSA obtained via the Dubois formula. Repletion events of B cells were defined as > 1% of CD19^+^ cells of total lymphocyte counts. The authors suggest to determine the BSA via the Mosteller formula, especially in patients with large height and weight, to avoid suboptimal dosing for patients with a high BSA. In the same study, age was not related to the level of B cell replenishment [107]. However, analyses in greater cohorts are needed to validate these findings as the question of age-dependent repletion is essential, especially regarding safety issues.

### Dosage and Application Forms

The essential question of the adequate dosage and dosing frequency of any of the anti-CD20 is still in the focus of discussions. At present, every anti-CD20 antibody has its own protocol with a definite dosage, which can vary considerably from one another: OCR is approved with 600 mg i.v. every 6 months, while UB is currently used at 450 mg every 12 weeks in phase III clinical studies. For i.v. RTX, no approved protocol exists, but it is used at 1000 mg every 6 months in most studies. On a mg-to-mg basis, RTX is thought to be three to five times less potent than an equal dose of OCR [108]. In contrast, OFA, the only s.c. anti-CD20 mab so far, is dosed at 20 mg s.c. every 4 weeks. As dosage is dependent on the application form, it is understandable that OFA is used in a lower dose in terms of mg compared to other anti-CD20 antibodies. However, knowing that B cell depletion and most likely also repletion show a dose-dependent effect, interesting observations can be made from comparing OFA and OCR. B cells are efficiently depleted from the blood with 20 mg s.c. OFA monthly. At the same time, a rapid repopulation of B cells after cessation of the OFA treatment is possible due to most probably incomplete depletion of B cells in lymphoid tissues [31]. In contrast, a dose of 600 mg OCR i.v. delays reconstitution of B cells, most likely due to a greater depletion of B cells in blood and LNs [29]. Interestingly, both antibodies and their respective dosage show an outstanding effect on relapse biology and focal MRI activity. This finding poses the question whether a low-dose treatment and hence a non-complete CD20^+^ cell depletion might be sufficient for the treatment of MS [74, 99]. Lower dosage and higher frequency intervals would improve the safety-risk profile of CD20 depleting drugs.

However, it is important to investigate not only the effect of anti-CD20 therapies concerning RAW, but also in regard to the inhibition of PIRA. Low-dose treatment seems to be highly efficient regarding the inhibition of relapses and RAW; however, it is not clear yet to what extent anti-CD20 drugs and the peripheral depletion of CD20^+^ cells can act on smoldering MS. Disease progression and brain volume loss proceed under anti-CD20 therapy [31]. This goes in line with the relatively moderate effect of anti-CD20 treatment shown in PPMS compared to the outstanding effects in RMS. Furthermore, novel imaging techniques indicate that part of the demyelination and the damage due to progression of disease is not visualized on conventional MRI [109]. Little is known about the actual effect of anti-CD20 treatment on intrathecal B cells. However, as intrathecal B cells are most likely the origin of abnormal production of Igs in the CNS, it might be necessary to also target these cells in order to silence smoldering MS and PIRA. On the one hand, levels of B cells in the CSF drop during treatment with anti-CD20 mabs. On the other hand, a post mortem analysis of individual cases of MS patients treated with RTX showed that lymphocytic inflammation in plaques, including CD20^+^ cells, is still present [110]. Interestingly, in a study in which RTX was administered i.v., only 0.1% of the RTX concentration on plasma levels reached the CSF [111]. However, in order to achieve intrathecal B cell depletion, the drug has to reach the CNS in a certain concentration. Investigations of exposure-stratified analysis of 24-week CDP in patients treated with OCR showed that a high concentration of the drug in the CNS and low levels of B cells have a positive influence on disability progression [112]. Intrathecal administration of RTX has, until now, only shown disappointing results. The drug is probably rapidly eliminated from the CSF through venous drainage and additional pharmacodynamic alterations in the CNS decrease its efficacy, concentration, and effector functions. Komori et al. [113] concluded that as long as the BBB is intact, CDC and ADCC are not potent mechanisms regarding intrathecal B cell depletion due to physiologically low levels of complement and low concentrations of cytotoxic NK cells in the CNS. Consequently, even further developed and more effective CD20 antibodies such as OCR, OFA and UB would likewise only show modest effects when administered directly into the CNS [114]. Novel mechanism or engineered modifications of antibody structures must be developed to achieve effective mechanism within the CNS when the BBB is undamaged.

A current phase III trial tests 600 mg OCR i.v. against 1200 mg and determines intrathecal B cell depletion to better understand whether a higher dose targets CNS demyelination and the smoldering part of MS more efficiently (NCT04544436). In this context, Hawker and colleagues [28] performed a subgroup analysis of PPMS patients treated with RTX who were younger than 51 years and had at least one GEL. They found that treatment effects were primarily driven by the rate of progression and dependent on the active inflammatory load. This observation goes in line with the abovementioned ORATORIO subgroup analysis, in which Wolinsky et al. [45] revealed that subjects with higher inflammatory load had a greater benefit of treatment. Nevertheless, it has to be mentioned that the trial was not powered for comparisons of prespecific subgroups.

To summarize, the answer regarding the ideal dosage of anti-CD20 mabs is most probably dependent on the dominant course of disease activity. In order to suppress relapse biology, low-dose treatment seems to be sufficient and reconstitution kinetics of peripheral B cells or even more precise memory B cells might be the parameter to adapt dosage and frequency of treatment to [74, 101, 115]. However, when it comes to targeting progression independent of relapses, high-dose treatment and the depletion of intrathecal B cells might be more efficient and relevant.

The abovementioned findings show the importance of differentiating accumulation of disease into RAW and PIRA as well as subdividing PPMS into different courses, depending on the level of disease activity as has been done in the new classification by Lublin et al. [1]. It is strongly suggested to use the classification of PPMS subdivided into “active and with progression,” “active but without progression,” “not active but with progression,” and “not active and without progression” as well as the concepts of RAW and PIRA for future studies.

### Biomarkers and Alternative Strategies to Target B Cells

Alternative B cell–depleting treatments such as anti-CD19 mabs and treatments targeting crucial key survival factors such as B cell activating factor (BAFF) and proliferation-induced ligand (APRIL) did not show equally promising clinical results. Still, some conclusions can be drawn. First, the differences in efficacy of CD19 and CD20 targeting treatments could indirectly hint at the role of CD20^+^ T cells in the pathogenesis or disease course of MS as these cells do not express CD19 on their cell surface and are hence not depleted with anti-CD19 mabs [92]. Second, anti-CD20 treatments do not target pro-B cells and late differentiated B cells in contrast to the abovementioned other B cell directed therapies. The elimination of plasmablasts and plasma cells is—contrary to expectations—most likely not beneficial for RMS patients [116]. A trial with atacicept, an antibody against BAFF and APRIL, was interrupted because the course of RMS disease worsened for unknown reasons. Presumably, this negative effect on the disease was mediated by an increasing number of memory B cells while regulatory B cell function was reduced [117]. Animal studies show that B cell depletion can also be counter-productive and ineffective in some contexts. From these opposing observations, the question arises whether pan B cell depletion is not beneficial in every context [11, 118]. In this regard, research is currently trying to find biomarkers to evaluate clinical benefit of anti-C20-depleting therapies and predictive markers to identify patients who will profit from this kind of therapy. A study called OBOE (Ocrelizumab Biomarker Outcome Evaluation) focusses on the longitudinal evaluation of CSF before and after OCR treatment (600 mg every 24 weeks) and is investigating to what extent OCR is capable to change CSF findings. So far, preliminary results in subjects with RMS show that OCR treatment led to a reduction of IgG in the CSF at week 12 and to a significantly higher reduction at week 52. When correlating these results to the detection of OCBs, a decrease in IgG OCBs could be seen and three out of 15 patients lost previous OCB positivity in week 52 [119]. Furthermore, pretreatment levels of neurofilament light chain (NfL) in serum and CSF correlated with each other and were associated to the total amount of lesions on brain MRI. OCR treatment led to a decrease of NfL levels in serum and CSF as well as to a reduction of B cells in the CSF [120]. These results must be further validated with long-term data including PPMS patients. The current data, however, suggest that OCR impacts intrathecal Ig production and NfL levels, two promising biomarkers of MS which are helpful in regard to progressive biology when MRI activity is not detectable.

The current efforts in exploring long-term safety issues of B cell depleting therapies and other lasting immunosuppressive therapies, especially with regard to higher infection risks, show the necessity for short-term reversible drugs in the treatment of MS. One promising approach are BTK inhibitors, which are so far known from and approved for the treatment of different B cell malignancies and some autoimmune diseases. BTK is an enzyme that belongs to the TEC family and that is mainly expressed on the haematopoietic system, except for T cells, NK cells, and plasma cells [121, 122]. Together with phosphatidylinositol 3-kinase isoform p110 delta (PI3Kδ), it plays a central role in adaptive immunity, as it is essential for the maturation and differentiation of B cells. However, it is also present on innate immune cells such as macrophages, dendritic cells, and microglia. The deficiency of BTK, known, for example, in the X-linked agammaglobulinemia disease, leads to very low levels of circulating Igs due to a lack of functional B cells.

The most studied BTK inhibitor is ibrutinib (PCI-32765), approved for CLL, Waldenström’s macroglobulinemia, mantle cell lymphoma (MCL) (second line), marginal zone lymphoma, and chronic graft-vs-host disease [123–127]. In recent years, the use of novel BTK inhibitors was extended to autoimmune diseases such as RA and SLE. In a study evaluating the effects of ibrutinib in patients with RA, BTK inhibition led to a lower synovial inflammation and a reduced infiltration of macrophages into the synovium. The treatment resulted in lower levels of cytokines as well as diminished levels of auto-reactive antibodies [128, 129]. In EAE, BTK inhibitors act as B cell and monocyte silencers, lowering cytokine production, impairing antigen presentation, and altering the composition of immune cells [130, 131]. As BTK inhibitors dampen B cell function, four BTK inhibitors are currently tested in phase I, II, or III clinical trials for the treatment of MS: evobrutinib (M2951, MSC2364447), tolebrutinib (PRN-2246, SAR442168), fenebrutinib (BDC-0853, RG7845), and BIIB091. The main advantages of BTK inhibitors are thought to be their rapid reversibility and their potential to penetrate the BBB [132]. The rapid reversibility is achieved by either irreversible binding but rapid de novo synthesis (evobrutinib, tolebrutinib), or by non-covalent binding to the kinase (fenebrutinib). Fenebrutinib is the most selective and potent targeting BTK inhibitor [133, 134]. Furthermore, BTK inhibitors are less likely to trigger antibody responses against the drug than anti-CD20 antibodies, and hence, they also trigger fewer allergic reactions. Due to superior selectivity over ibrutinib, they are all thought to have a better overall tolerability.

Evobrutinib, fenebrunib, and tolebrunitib showed clinical benefit in phase II clinical trials and will further be investigated in phase III clinical trials in RMS and PPMS. While evobrutinib (NCT04338022, NCT04338061) and tolebrutinib (NCT04410978, NCT04410991) are tested against TERI in subjects with RMS, fenebrutinib is investigated in both RMS patients (NCT04586023, NCT04586010) and subjects with PPMS (NCT04544449).

## Conclusions

The remarkable results of anti-CD20 treatment in patients with RMS and PPMS have stirred up the field of MS research. Anti-CD20 antibodies represent a highly efficient therapy in MS patients regarding the prevention of clinical relapses and to a certain extent also disability progression. Additionally, the research on anti-CD20 drugs has questioned the long-existing paradigm of T cells being the key mediator in the pathogenesis of MS. In recent years, much effort was laid on the development of new anti-CD20 antibodies. Until now, two novel, partly (OCR) and fully human (OFA) anti-CD20 mabs have shown clinical and radiological benefits for patients with RMS and PPMS and have thus been approved by the FDA for the treatment of MS. OCR takes up a special role as it is the first and so far only approved therapy for patients with PPMS. OFA is the only s.c. administered anti-CD20 drug.

As MS is a chronic, and until now not curable disease, it is important to think in the long term when treating MS patients. Therefore, it is necessary to find a balance between an acceptable risk associated with a treatment and the prevention of rebound activity and clinical progression. Anti-CD20 agents have a favorable short-term safety profile with the main adverse effect being mild to moderate IRRs. However, in some patients, B cell depletion led to a decrease of Igs as a secondary effect of insufficient antibody production, which might be associated with an increased risk of infections. Furthermore, cases of higher frequencies of malignancies have been reported in anti-CD20-treated groups compared to placebo. However, the incidence was not above the expected overall rate for malignancies. Although no cases of PML were reported in the pivotal studies of RTX, OCR, OFA, and UB, isolated reports mentioned the occurrence of carryover PML under anti-CD20 therapy. In order to get a better picture of the actual risk and safety profile of any drug, it is important to record long-term real-world data. Extension phases of admission studies are a good approach already. However, it is also necessary to evaluate efficiency and safety of a drug in patients not fulfilling the inclusion criteria of most studies to prove that the risk profile of the broad range of MS patients is not underestimated. On top of this, the development of antidrug antibodies and their effects on the efficacy of anti-CD20 depletion should be studied carefully.

Captivatingly, recent studies raise the question whether near-complete depletion of circulating CD20-positive cells is necessary for a relapse-free state of disease or whether low-dose or higher-interval treatment may result in a better safety to risk profile with a suppression of disease activity that is still satisfying. This idea is supported by the observation that B cell levels were still very low at 24 weeks after a 600 mg i.v. administered OCR dose and that a 20 mg dose of s.c. applied OFA every 12 weeks led to an incomplete B cell depletion with a still high and sufficient capacity of suppressing new GEL in MRI. At the same time, it is still unclear to what extent anti-CD20 therapies can influence smoldering MS, and if high-dose treatment can be more beneficial regarding inhibition of progression independent of relapses. In the future, it will be necessary to answer the question whether high-dose episodic treatment or continuous treatment is superior in terms of the benefit-to-risk ratio.

Analyses of subgroups of the pivotal studies have revealed interesting aspects on disease course, progression rate, and treatment strategies of MS. First, evidence suggests that anti-CD20 therapies with a relatively good risk-to-benefit are a possible first-line therapy of newly diagnosed, treatment-naïve patients instead of the traditional escalation approach. Suppressing relapse biology and focal MRI activity early will give new insights into the effects of relapses on disease progression. Second, the analyses show the importance of differentiating between two therapy approaches—namely prophylaxis of lesions and inhibition of progression. Anti-CD20 treatment shows outstanding results on targeting relapses and the occurrence of new GEL, but their effects on progression remains unclear. This observation goes in line with possibly higher benefit for PPMS patients with active disease and progression than for subjects with a non-active form of disease and progression. In order to classify deterioration of disease more precisely, the new concept of CDA subdivided into RAW and PIRA should be applied in future studies.

A time-delated repletion of B cells takes place after cessation of treatment because CD20 depletion targets a broad range of B cells, but spares plasma cells, pro B-cells, and stem cells. The recovery of B cells is beneficial regarding long-term safety concerns; however, recent studies suggest the repleting B cell pool to possess a more pro-inflammatory B cell phenotype. With regard to potential differences between the different anti-CD20 mabs in the reconstitution kinetics of B cells, more research is needed. Furthermore, special attention is drawn to the depletion of memory B cells and their role in regard to relapse biology. Another cell population, which is currently discussed to have a potential impact on the clinical benefit of anti-CD20 therapies, are CD20^+^ T cells. They only represent 20% of CD20^+^ cells, and their contribution to the effect of anti-CD20 drugs is not yet clarified.

As a next pivotal step in the treatment of MS, future therapies should aim at developing therapies that more selectively silence pathogenic B cell function but spare out non-pathogenic regulatory B cell properties instead of promoting near pan B cell depletion as caused by anti-CD20 mabs. In recent years, evidence has accumulated that B cells have the potential to positively regulate immunity. However, this regulatory component has not yet been clearly associated to one specific subtype of B cells in humans. This fact may indicate that regulatory functions are not strictly linked to a particular cellular subtype but rather are context dependent. To achieve a more selective and reversible B cell–targeting treatment, more research should be addressed to identify how pathogenic B cell function can be eliminated while preserving regulatory B cell functions.

A new approach, which is especially interesting regarding short-term reversibility and application form are BTK inhibitors. These inhibitors are currently tested in phase III studies in patients with RMS and PPMS. BTK inhibitors are bioavailable upon oral treatment and are thought to target both acute and chronic inflammation through their dual mechanism of affecting the adaptive (B cells) and innate immunity (monocytes and microglia). After first promising results in phase II and already launched phase III clinical trials, in a few years, BTK inhibitors might represent a new, reversible B cell targeting therapy for the treatment of MS.

To conclude, the remarkable effects of anti-CD20 antibodies in patients with RMS have not only introduced a new and highly efficient approach in the treatment of MS but have also reshaped the understanding of the pathogenesis of MS. At last, factors including efficacy, safety, feasibility, costs, long-term treatment strategies, and personal preference on the application method will determine the use of the two approved antibodies OCR and OFA and future anti-CD20 antibodies in the upcoming years.

## Supplementary Information

Below is the link to the electronic supplementary material.

Supplementary file1 (PDF 463 KB)

Supplementary file2 (PDF 508 KB)

Supplementary file3 (PDF 471 KB)
